# Applications and Potential of In Silico Approaches for Psychedelic Chemistry

**DOI:** 10.3390/molecules28165966

**Published:** 2023-08-09

**Authors:** Sedat Karabulut, Harpreet Kaur, James W. Gauld

**Affiliations:** 1 Department of Chemistry and Biochemistry, University of Windsor, Windsor, ON N9B 3P4, Canada; sedat.karabulut@uwindsor.ca; 2 Pharmala Biotech, 82 Richmond Street E, Toronto, ON M5C 1P1, Canada; harpreet@pharmala.ca

**Keywords:** Central Nervous System, computational modeling, in silico, serotonin, dopamine, MDMA, psychedelics, molecular dynamics, docking, quantum mechanics/molecular mechanics (QM/MM), Structure-Activity Relationship (SAR), machine learning, artificial intelligence

## Abstract

Molecular-level investigations of the Central Nervous System have been revolutionized by the development of computational methods, computing power, and capacity advances. These techniques have enabled researchers to analyze large amounts of data from various sources, including genomics, in vivo, and in vitro drug tests. In this review, we explore how computational methods and informatics have contributed to our understanding of mental health disorders and the development of novel drugs for neurological diseases, with a special focus on the emerging field of psychedelics. In addition, the use of state-of-the-art computational methods to predict the potential of drug compounds and bioinformatic tools to integrate disparate data sources to create predictive models is also discussed. Furthermore, the challenges associated with these methods, such as the need for large datasets and the diversity of in vitro data, are explored. Overall, this review highlights the immense potential of computational methods and informatics in Central Nervous System research and underscores the need for continued development and refinement of these techniques and more inclusion of Quantitative Structure-Activity Relationships (QSARs).

## 1. Introduction

Mental health is one of the fastest-growing global health challenges. For instance, the two most common mental disorders are anxiety and depression, which cost the global economy US$1 trillion per year, with total costs projected to rise to US$6 trillion by 2030 [1]. A fundamental approach to treating such health conditions is to use pharmaceutical interventions, such as SSRIs (Selective Serotonin Reuptake Inhibitors), SNRIs (Serotonin-Norepinephrine Reuptake Inhibitors), and psychotherapy, to improve an individual’s quality of life. The rapidly rising costs underscore the ever-growing need for new, modified, and more effective therapeutics. This, in turn, requires a greater understanding of critical components of the Central Nervous System (CNS), specifically at the biomolecular level.

**Figure 1:**
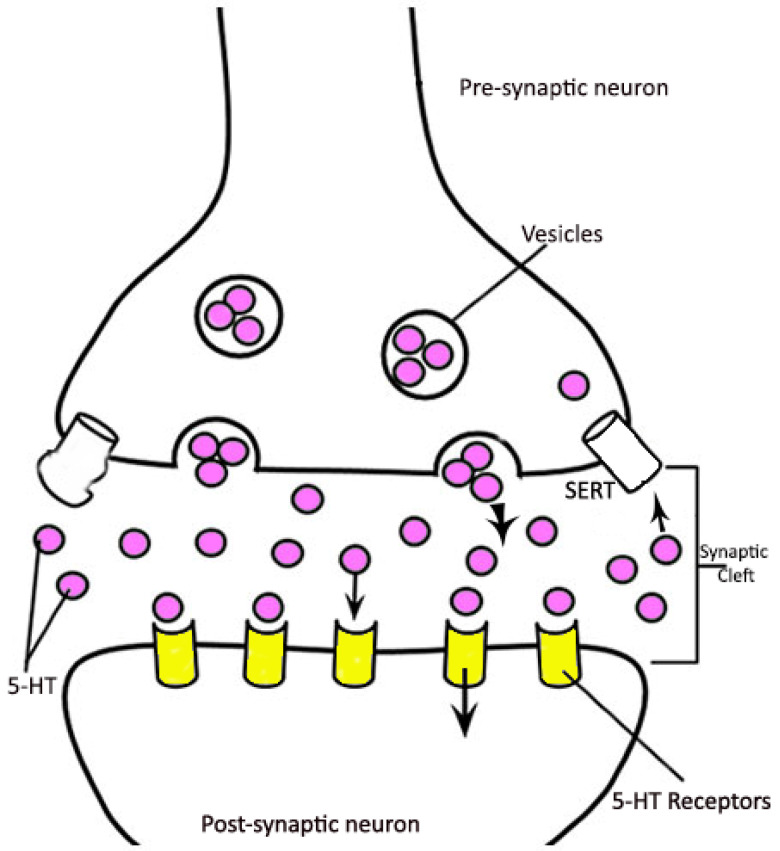
A general overview of monoamine transportation.

The CNS processes information and delivers it to the peripheral nervous system. This neurotransmission process occurs through signal conduction via synapses from one neuron to another [2]. It plays a critical role in, for example, signaling that coordinates muscles (including cardiac) and proper bodily fluid secretions and organ functions. Chemically, the penetration of calcium into the synaptic terminal causes synaptic vesicles, which are mainly responsible for the storage of neurotransmitters, to release them into the synaptic cleft (Figure 1). The neurotransmitters then bind to receptors on the postsynaptic neuron to create the electrical response [3], enabling neurotransmission. The biogenic monoamine catecholamines (e.g., dopamine, norepinephrine, and epinephrine) and serotonin are functionally essential brain neurotransmitters. Dopamine (DA) and serotonin (5-HT) have leading roles in the brain, including the control of locomotion, mood alteration, and behavior [4,5]. The concentration of the neurotransmitters in the brain (more specifically, their relative proportions in a vesicle, synapse, and synaptic cleft) relates to the biochemical pathology of numerous neurological disorders, including depression, anxiety, and post-traumatic stress disorder (PTSD) [6]. Since there is a direct relationship between the chemical processes in the CNS and these diseases, a deeper understanding of the molecular mechanism would enhance prevention and treatment [7,8].

The CNS is complex, with multiple components (e.g., transporters, enzymes, and receptors) acting in a combinatorial way to produce signals, with each key step triggered by a molecular-level interaction (e.g., drug/neurotransmitter-protein or protein-protein). Central to understanding such interactions is obtaining greater insight into their nature and structure. However, studies of the Structure-Activity Relationship (SAR) of neurotransmitters and therapeutic drugs have previously proven to be challenging. For example, the more active enantiomer of hallucinogenic amphetamines is generally “R”, but for a few compounds (generally “Entactogens”) like 3,4-Methylenedioxymetamphetmine (MDMA), it is “S”. In addition, while attaching alkyls to its alpha carbon and/or nitrogen generally abolishes the activity of amphetamines, this is not the case for MDMA [9].

**Figure 2:**
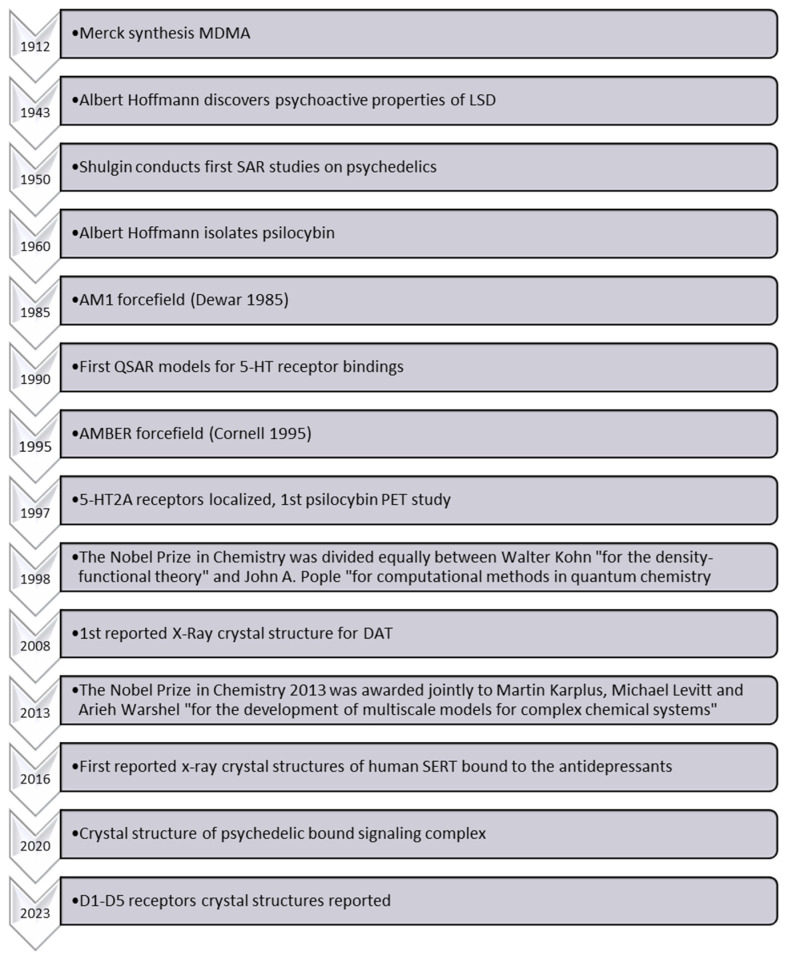
Timeline of key events in computational psychedelic sciences [20,21].

The SAR of MDMA, also known as ecstasy, differs significantly from that of amphetamines. Indeed, it exhibits unique pharmacological effects, particularly pronounced serotonin-releasing properties and entactogenic effects, which thus distinguish it from other amphetamines [9]. However, the precise molecular mechanisms underlying its distinctive SAR and serotonin release remain largely elusive [10,11,12,13,14]. While various studies have explored the behavioral and neurochemical effects of MDMA, the complexity of its interactions with biological targets (transporters, receptors, and enzymes) hampers a comprehensive understanding of its precise mode of action. Despite Shulgin reporting experimental SAR data on MDMA and other psychedelic compounds almost 50 years ago [15], a greater understanding of MDMA’s biochemical interactions and effects is also inhibited by the scarcity of reliable experimental evidence elucidating these phenomena at the molecular level. Since Shulgin’s publications [15,16,17], several investigations have further enhanced our knowledge of such compounds and their bioactivity (Figure 2). Indeed, the diversity of reported in vitro data, the occurrence of multiple drug targets (combination of multiple receptor signals) for a single behavioral outcome, the limited availability of suitable experimental techniques, and ethical considerations regarding human studies further complicate the acquisition of reliable molecular-level data [18,19]. Therefore, further research utilizing cutting-edge methodologies, such as advanced imaging techniques and computational modeling, coupled with well-designed experiments, is crucial to unraveling the intricate mechanisms underlying MDMA’s SAR and its specific serotonin release mechanism, ultimately enhancing our understanding of its unique pharmacological properties.

Computational chemistry is an established and powerful approach for reliably and accurately investigating and predicting the chemistry and properties of (bio)chemical systems. As highlighted in Figure 2, this is because there have also been considerable advances in the power and applicability of such in silico methods, enabling their application to such diverse areas as the molecular basis of cancer biochemistry to virology, including COVID-19 [22]. Indeed, computational modeling and simulations of possible therapeutics have shown great potential to facilitate drug discovery and development [23]. Furthermore, in silico methods have enabled more significant insights to aid in rationalizing or interpreting trends, Structure-Activity Relationships, and the identification of some mechanisms of the CNS [17,24,25,26,27].

As noted above, the first SAR studies on psychedelic drugs appeared in the 1950s, primarily focusing on the drug compounds’ active enantiomers [28,29,30]. Meanwhile, the effects of structure and stereoisomerism on psychedelic activity were extensively discussed in the “Quantitative Structure-Activity Relationships (QSAR) of Narcotic Analgesics, Narcotic Antagonists, and Hallucinogens Conference” in 1978 [11]. For example, it has been reported that for lysergic acid diethylamide (LSD) [31], 4-bromo-2,5-dimethoxyphenylisopropylamine (DOB) [30], and 4-methyl-2,5-dimethoxyphenylisopropylamine (DOM, STP) [32], the active enantiomer is the “R” configuration. However, for other potential drugs such as MDMA, the more active optical isomer is the “S” enantiomer.

**Figure 3:**
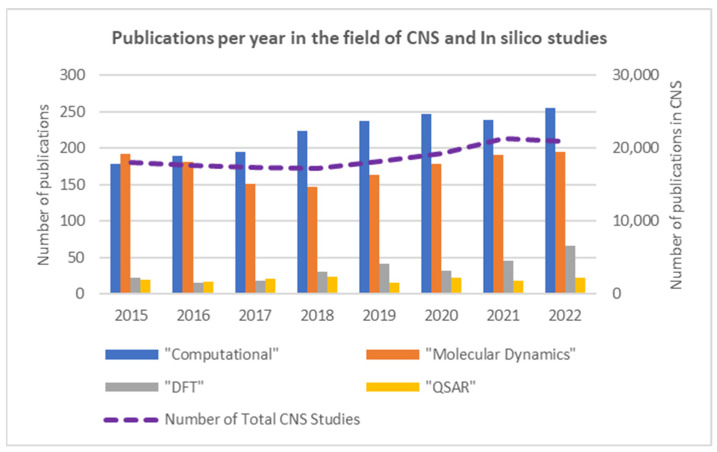
The purple dashed line represents the total number of publication hits, for 2015–2022, on Scopus for studies that also contained one or more of the terms “CNS”, “serotonin”, “dopamine”, or “norepinephrine” as of 20 March 2023 (right ordinate). Each column, within a given year, indicates the number of publications (within the same above search criteria, left ordinate) that also include the related in silico terms “Computational”, “DFT”, “Molecular Dynamics”, and/or “QSAR”.

However, as shown in Figure 3, the uptake and application of atomistic or electronic in silico methods to the molecular-level study of the CNS have not been as significant: in silico publications account for only 2.3% of all CNS publications. Its utility and impact can be further enhanced when complementarily applied in a multi-scale approach and/or trained or coupled with experimental data. For the latter, it is notable that an ever-increasing number of experimental in vitro studies on the CNS [33,34,35,36,37,38] could provide such invaluable data. Thus, well-considered and implemented computational tools present a powerful approach with the potential to greatly enhance and expand our understanding of the CNS, including atomic-level insights into the SAR of small molecule (e.g., drug)-transporter/receptor interactions. Indeed, in a 2018 review, Makhouri and Ghasemi highlighted in silico drug studies for neurodegenerative diseases [39] and concluded that such methods are powerful tools for target identification, discovery, and optimization of drug candidate molecules.

DFT (Density Functional Theory) methods have emerged as the method of choice for studying biochemical systems, offering reliable and accurate insights at the electronic level. This is because they are computationally cheaper than wavefunction-based electron-correlated methods yet are able to provide reliable and accurate insights. This has enabled the study of large biomolecular systems that would otherwise be intractable with more computationally demanding approaches. In the context of biochemical systems, researchers can explore their intricate interplay of atoms and electrons reliably and accurately, providing valuable information about molecular geometries, electronic energies, and reaction mechanisms [37,38,39]. As a result, such approaches have significantly contributed to our understanding of fundamental biological processes and facilitated the design of novel drugs and biomaterials [40,41].

In this present review, we aim to provide a timely summary of the current state of affairs with regards to the use of in silico methods (specifically docking, molecular dynamics (MD), quantum mechanics (QM), chemical clusters, quantum mechanics/molecular mechanics (QM/MM), and informatics (SAR and QSAR)) for the study of the CNS and, by extension, also help highlight areas where they might be able to contribute further.

## 2. Computational Modeling

**Figure 4:**
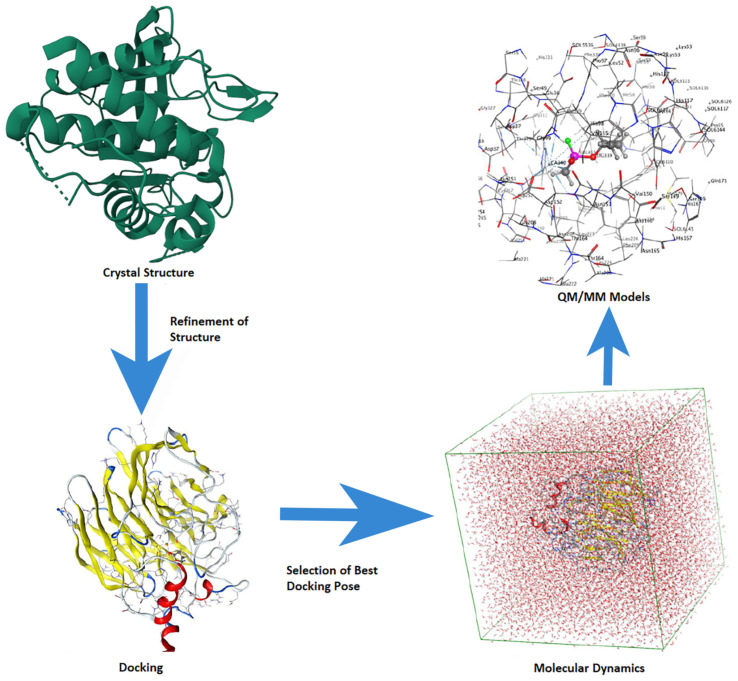
A multi-scale computational approach is generally used for enzyme mechanism studies.

The application and utility of computational chemistry in (bio)molecular modeling to help explain experimental observations and guide future studies are well established. The increasing power of computers coupled with computational method development has greatly expanded the potential of this approach for many fields, including the drug discovery process and computer-aided drug design (CADD) [40]. Indeed, there is now quite a broad range of methods that one could use, depending on the goal, including docking, pharmacophore design, structure- and fragment-based drug design, MD simulations, QM, and hybrid quantum mechanics/molecular mechanics (QM/MM). With any single approach, there are, of course, compromises to be managed. For example, while Docking and MD simulations can be applied to very large systems (e.g., protein-ligand and protein-protein complexes), they do not usually allow one to obtain explicit electronic-level insights. In contrast, quantum mechanical methods can provide electronic-level information. However, correlated wavefunction-based methods can only be applied to chemical models of very limited size (e.g., approximately 30 heavy atoms). Meanwhile, density functional (DFT) methods can be applied to chemical models of up to 200–300 heavy atoms [41]. Newer methods such as QM/MM aim to address this gap by allowing us to divide a chemical system into layers: the core region (high layer) in which reactions, etc. occur is modeled at a higher level of theory, while the surrounding layer(s) (the low layer(s)) are modeled at a lower level of theory (e.g., MM). Such an approach aims to complementarily exploit the strengths of the various methods to allow larger systems to be studied. However, increasingly, computational studies use a multi-scale approach; several methods are complementary to obtain even greater insights than any one method could achieve on its own, as visualized in Figure 4. There are numerous general reviews on the use of such methods for studying, for instance, enzymes. These approaches are extensively used in academia and industry, including pharmaceutical companies, and they can reduce costs and accelerate the (bio)molecular target identification process [42].

Computational studies may require the structure of, for instance, a target protein or additional experimentally derived data. Homology modeling has made great strides in recent years [43]. A detailed analysis and comparison of current known monoamine transporter (MAT) crystal structures and homology modeling focused on dopamine transporters (DAT), a major class of MATs, was reported by Jones et al. [43]. They also noted that the sequence similarity of MATs (i.e., serotonin (SERT), dopamine (DAT), and norepinephrine (NET) transporters), *Drosophila melanogaster* (dDAT), and human (hDAT) DATs were 40% and 50%, respectively. In general, homology structure prediction is more accurate and reliable the greater the similarity between the sequences of the ‘unknown’ target protein and those with known structures. Thus, the availability of good experimental crystal structures is often an essential initial step for providing a good template for further computational-based investigations. However, this can also present challenges beyond finding a sufficiently well-resolved structure. For instance, the crystal structures of hDAT, NET, and SERT with releasing drugs and DAT with occupied allosteric sites have not been experimentally reported.

Historically, computational investigations into the CNS began in the early 1990s. For instance, in 1991, Dahl et al. [44] performed a detailed MD simulation investigation of the D2 dopamine receptor. More specifically, based on its sequence, they used a homology modeling approach to build a 3D structure of the target receptor and subsequently investigated various binding properties. They concluded that protonated agonists may induce conformational changes in the D2 receptor and that an aspartate (Asp80) and asparagine (Asn390) residue had critical roles in binding. The extent to which the power and potential of computational approaches have grown is illustrated by the highly cited study by Keiser et al. in 2009 [45]. They reported the binding affinities of 3665 Food and Drug Administration (FDA)-approved CNS drugs against more than 1400 targets. Meanwhile, in 2009, Kaufmann et al. used the Rosetta Ligand docking program to identify and determine the binding site of 5-HT on SERT [46]. In 2011, they used the same approach to also study S-citalopram binding with human SERT and reported that a tyrosine (Tyr95) and a glutamate (Glu344) residue are important for binding [47].

### 2.1. Docking

Docking as a tool to both help identify possible binding sites and potential ligands has become a very common and useful approach, as evidenced in part by the number of programs now available as well as the publications in which it has been used. Herein, we will highlight several examples of how it has been used within the context of studies on the CNS.

The activity of monoamines is mediated through the activation of one or more members of a large family of receptor proteins [48], which can be thought of as front-line members of the CNS. High-resolution 3D X-Ray crystal structures are now available for most receptors, especially 5-HT receptors. In 2013, Wang et al. reported a detailed study, now highly cited, that also at least in part involved Docking on 5-HT receptors [49], specifically a human 5-HT1B G protein-coupled receptor (GPCR) bound to a diverse variety of ligands, including agonists, antimigraine medications, ergotamine, and dihydroergotamine. Furthermore, they could also compare the pharmacological binding properties of 5-HT1B/5-HT2B and human 5-HT1B/rodent 5-HT1B. It is noted that recently, several experimental and computational studies have greatly expanded and enhanced our knowledge and information about these receptors and thus potentially provided new opportunities to better understand their ligand binding properties [50,51,52]. The latest crystallographic data on dopamine receptors was published recently, which reports cryogenic electron microscopy structures of all five subtypes of human dopamine receptors (D1–D5) in complex with G protein [53]. It is expected that this report will accelerate computational research on dopamine receptors.

In addition, it is also noted that several previous reviews have touched upon or focused on computational contributions to the field of drug design and, in some cases, therapeutic drugs for CNS diseases [39,54]. These also highlighted challenges faced at that time, including that some therapeutic-related phenomena such as protein-protein interactions and/or protein-DNA interactions were formidable problems in part since the size of the systems was perhaps beyond, or at best at the limits, of the then generally available computational power [54]. However, advances in both software and hardware have led to faster and more economically sustainable drug discovery processes. Furthermore, there have been significant developments in the generation of chemical libraries, often containing data on billions of compounds [38,55], as well as specific databases for existing or putative natural compounds. As a result, in silico docking, which can be coupled with other methods (e.g., MD, QSAR, etc.), has greatly advanced in just a few years as a promising approach for the screening of chemical space and subsequent design of a possible therapeutic compound for a given disease [56]. In 2019, Lyu et al. [55] examined possible ligands or leads for binding AmpC β-lactamase and the D4 dopamine receptor. They reported docking scores for 170 million compounds. The top-ranked compounds were synthesized, and it was concluded that the in silico docking results were in good agreement with the subsequent experimental results, thus highlighting both the chemical space that can now be computationally searched as well as the power of well-considered synergistic computational/experimental approaches. Where structures may not be available, docking can be used, even complementarily with other methods. For example, Hofmaier et al. [57] used homology modeling, docking, and DFT calculations, along with experiments, to identify the residues of the central binding sites of MATs involved in binding neutral and protonated levamisole. They concluded that levamisole, as a cocaine adulterant itself, directly inhibits the neurotransmitters DAT, SERT, and NET. Furthermore, they found that the levamisole metabolite aminorex has a cocaine-like effect on DAT and NET and an amphetamine-like effect on SERT.

The recently published research article focused on SERT, a crucial protein involved in the removal of serotonin from synapses and the target of many antidepressant drugs. The study aimed to investigate the inward-open conformation of SERT, which is not typically targeted by known inhibitors but has shown promise for novel therapeutic interventions. Through an extensive computational docking approach, the researchers screened over 200 million small molecules and identified two potent inhibitors that selectively stabilize an outward-closed state of SERT. These compounds exhibited remarkable activity in mouse behavioral assays, demonstrating anxiolytic and antidepressant properties superior to fluoxetine (Prozac). Furthermore, one of the inhibitors displayed significant potential for reversing morphine withdrawal effects. The findings provide valuable insights into the structural dynamics of SERT and offer promising avenues for the development of new drugs targeting the inward-open state [58]. The cryo-EM structure of one inhibitor bound to SERT validated the predicted geometry, enhancing our understanding of the molecular interactions underlying its therapeutic effects. These findings hold great significance in the field of mental health research, particularly in the design of more effective antidepressant and substance withdrawal treatments.

### 2.2. Molecular Dynamics (MD) Simulations

Other challenges that therapeutic drug design can encounter beyond simply drug binding are binding site identification and composition variability, conformational dynamics of the target, and their mechanism(s). For example, for the bacterial leucine transporter (LeuT) complexed with clomipramine, there are presently over 100 experimentally determined structures or computational models published, which also highlight a substantial degree of variability in hMATS-ligand recognition [59]. Recently, Xue et al. reviewed progress in the field’s understanding of the binding and mechanism of hMATs inhibitors within the central and/or allosteric sites [59]. They also performed extensive computational SAR and selectivity data for hit/lead compounds for binding hMATs, which were experimentally evaluated by in vitro and in vivo experiments. Meanwhile, Navratna and Gouaux considered the structures and mechanisms of eukaryotic Neurotransmitter Sodium Symporters (NSSs), specifically the molecular basis for ligand recognition and their transport mechanism [60]. As part of this, they also highlighted the important role such computational approaches as homology modeling, MD simulations, and ligand binding modeling have played in understanding the properties and roles of these and related transporters, including identifying possible binding sites for activity modulators (e.g., cholesterol). In doing so, it also illustrated the power of such approaches in that one can choose to model the interaction and binding of a single ligand in a preselected position or region. Similarly, Cheng and Bahar [61] have discussed progress in the elucidation of the structural dynamics of MATs and their conformational landscape and transitions, as well as allosteric regulation mechanisms [61]. In particular, it highlights the role molecular modeling, along with experimental studies, can play in elucidating the conformational behavior of a target throughout its role (e.g., binding, unbinding, etc.). In addition, in some cases, full-atomic microsecond MD simulations have shown that ligand binding alters the conformation of the target transporter. Despite such progress, a complete understanding of the function and mechanistic details of allosteric binding sites on MATs remains elusive, as does the mechanism of ligand and drug transportation, as well as the role of ions [14]. However, the above highlights the power and ability of computational approaches to study ligand-target binding in a static sense but also to go beyond and provide insights into the dynamic nature of biological targets and processes.

Several studies have examined the mechanistic details of MATs within the context of their roles in the CNS [12,14]. For instance, recently Islas et al. [12] performed an extensive computational study in which they complementarily applied induced fit docking, Monte Carlo simulations, pharmacophore modeling, and more to investigate the binding of 3,4-methylenedioxy-methamphetamine (MDMA) and entactogen analogs (MDA: 3,4-methylenedioxy-amphetamine; MDAI: 5,6-methylenedixoy-2-amino-indane; MBDB: 3,4-methylenedioxy-*N*-methyl-α-ethylphenylethylamine) with hSERT. MDMA, for instance, is used to treat various mental health disorders, such as post-traumatic stress disorder. Importantly, they obtained more significant insights into the binding of MDMA within the central binding site, an allosteric site, and the residues with which it interacts. From there, they could ascertain the possible roles and frequencies of ligand-residue interactions such as π-π and hydrogen bonds. In addition, they found that the MDMA could sample several possible binding positions and how it might migrate into or out of the central binding site. Notably, they also compared their computed interaction energies with experimental activities and showed they were correlated, thus helping to verify the reliability of their results. Yang and Gouaux [14] have experimentally investigated the molecular mechanism of ion-coupled serotonin reuptake by hSERT, with some standard modeling used to help refine the structures obtained. More specifically, they reported multiple molecular structures of hSERT in its apo state and with ligand bound. As a result, they could show detailed conformational changes of the central and allosteric sites during the transportation of 5-HT. They also suggested that during transportation, penetration of 5-HT between the allosteric and central sites is possible [14]. Unfortunately, especially considering the results of Islas et al. [12], no such data for MDMA, MDMA-like compounds, or commercial amphetamines is yet available. Future in silico studies may help elucidate this new transportation dynamism for ligands. Furthermore, while MDMA is a monoamine reuptake inhibitor and a releaser, there are presently few or no published studies (experimental or computational) on its reuptake inhibition and especially its release mechanisms.

### 2.3. QM-Cluster and QM/MM

**Figure 5:**
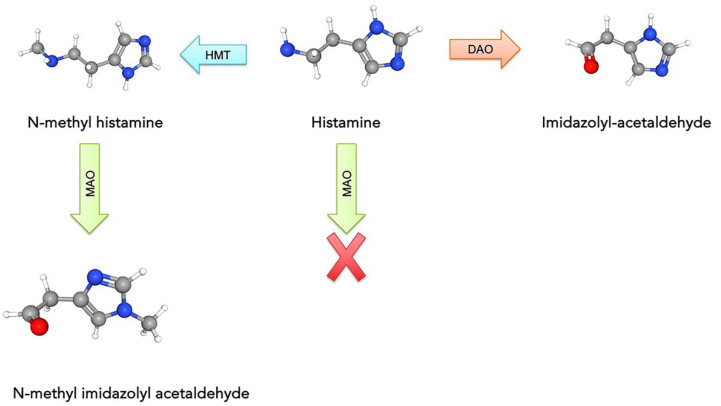
Metabolic degradation of histamine (HIS) to the corresponding aldehyde by MAO, but only after HIS is converted to *N*-methylhistamine (NMH) with histamine methyltransferase (HMT).

The above-mentioned studies have predominantly used Docking, MD simulations, and other general property computational methods to examine ligand binding with critical proteins and receptors of the CNS. However, one can also use more complex electronic-level QM methods. For instance, Marsavelski and Vianello computationally investigated the selectivity of monoamine oxidase-B (MAO-B) against histamine (HIS) and *N*-methyl histamine (NMH) [62]. Inhibition of this enzyme has been shown to decrease the degradation of the important signaling agent dopamine and help treat some symptoms in those with Parkinson’s disease. Computationally, they applied MD simulations and QM-chemical cluster methods and computed binding free energies using MM-PBSA. The experimentally observed selectivity of MAO-B for the *N*-methylated derivative of histamine over histamine itself has presented a challenge. More specifically, HIS cannot be reacted upon by MAO-B until it has undergone methylation of its imidazole ring by another enzyme (histamine-*N*-methyltransferase: HMT) or oxidative deamination of the primary amino group by diamine oxidase (DAO) (Figure 5). Using their multiscale computational approach, the authors were able to show that when bound, NMH is less flexible within the active site due to its higher tendency to form rigid conformations via hydrogen bonds with active site residues. This constrains NMH to a more reaction-feasible conformation within the context of the MAO-B mechanism. They were also able to obtain insights into the role of *N*-methylation on the strength of such interactions and show that binding is also enhanced via the hydrophobicity of the imidazole ring and the positioning and nature of several hydrophobic active site residues (Leu171, Leu328, and Ile199). Notably, their computed difference in activation energy for HIS and NMH of 2.6 kcal/mol was in very good agreement with the experimental value of 1.4 kcal/mol. This also highlights the accuracy and reliability achieved in well-considered and performed computational investigations.

The hybrid QM/MM method has become the default approach for studying the properties and mechanisms of biochemical systems such as enzymes. In commonly used two-layer QM/MM approaches, the high layer is the reactive region where bonds are made or broken, or the region containing the chemistry of interest. This is then modeled using a higher level of theory, which for biochemical systems is often DFT. The surrounding region, the low layer, is then modeled at a lower and thus computationally cheaper level of theory, commonly molecular mechanics (MM) [34]. This also enables the user to take advantage of the strengths of each method: a well-chosen DFT method can reliably and accurately describe electron-level changes such as bond formation or interactions. Meanwhile, MM methods can provide, for instance, good descriptions of long-range effects and sterics and more nuanced modeling of the surroundings and environment in which an active or binding site exists. While this allows one to study much larger systems than possible via QM alone, it also allows for detailed analysis of ligand binding or predicting intramolecular interactions. By dividing the system into layers, it can provide a compromise between computational cost and accuracy.

**Figure 6:**
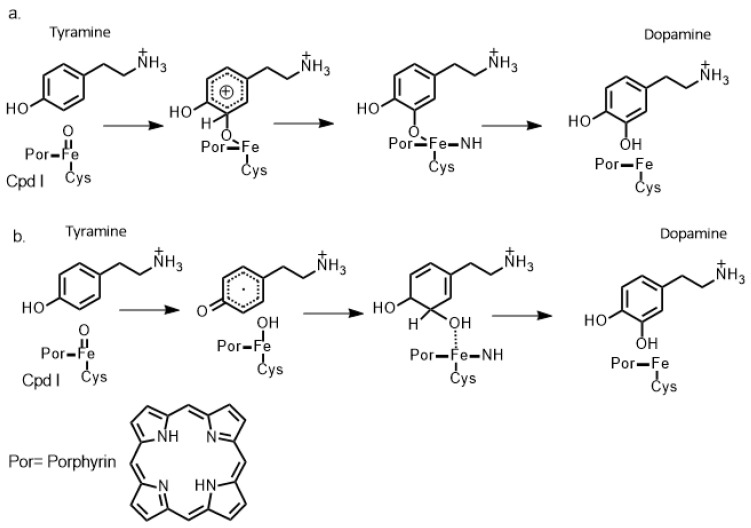
Schematic illustration of two proposed mechanisms of aromatic hydroxylation of Tyramine by Cpd 1, as proposed via (**a**) a Meisenheimer-complex mechanism and (**b**) a radical [65,66].

Indeed, QM/MM methods have been widely employed in recent years to study chemical processes such as enzyme-inhibitor interactions [39]. With regards to CNS-relevant studies, Kamachi et al. [63] and Yoshizawa, K. [64] used homology modeling to construct suitable protein templates and subsequent QM/MM studies (as well as some QM-cluster studies) to investigate the catalytic mechanism of dopamine-monooxygenase (DBM). Importantly, the latter enzyme catalyzes the oxidative conversion of dopamine to norepinephrine. They were able to use such approaches to discern the roles of key active site residues in terms of binding, structure, and catalysis—information that is also critical for therapeutic drug design. In addition, they also reported insights into the reactions and differences of the copper-superoxo, -hydroperoxo, and -oxo species. For instance, Kamachi et al. [63] concluded that copper-superoxo, -hydroperoxo, and -oxo species can detach a hydrogen atom at the benzylic position of dopamine with activation energies of 17, 40, and 4 kcal/mol, respectively [63]. A multiscale computational approach was also used by Schyman et al. [65] to investigate the important Cytochrome P450 enzyme (CYP2D6) and its catalytic mechanism of dopamine formation (Figure 6). More specifically, they used a multiscale approach of short (a few nanoseconds) MD simulations to obtain solvated equilibrated structures that were then used as templates for further QM-cluster and QM/MM calculations. The enzyme is involved in various hydroxylation reactions, including dopamine synthesis. Their results suggested that in the case of dopamine synthesis, instead of following a traditional Meisenheimer-complex mechanism, the mechanism instead involves an initial hydrogen atom transfer from the phenolic hydroxyl group of the initial tyramine substrate to the iron-oxo of the π-cation radical ferryl-oxo compound (Cpd I) [66]. This is then followed by a ring π-radical rebound that eventually leads to the formation of the desired dopamine product via a keto-enol tautomerization (see Figure 6).

Above are highlighted a broad range of successful applications of common computational chemistry methods (e.g., Docking, MD, QM-cluster, and QM/MM). Each class of method represents a significant area of application on its own, in addition to the increasingly applied multi-scale approaches. It is noted that for each, there are many recent thorough and detailed reviews [42,67]. As highlighted, the choice of method(s) is in part dependent on the properties and/or chemistry to be investigated. Additional factors include the available computational resources; the larger the chemical model and/or the more high-level the method, the greater the computational time and resources required. As with any computational approach, one way we can gain confidence in a particular method’s ability to provide accurate and reliable results is to examine and/or review its abilities for related systems. This is further enhanced where experimental data is available for comparison (e.g., structures, thermochemical values). Clearly, with the methods and resources increasingly available today, the range of chemical problems that we can accurately and reliably tackle is also increasing.

## 3. Structure-Activity Relationship (SAR) and Informatics

With advances in biotechnology-related experimental techniques, a large amount of experimental data has become available that can be exploited for modeling. Indeed, understanding the mechanisms, related pathways, and gene expression changes related to a range of mental illnesses (e.g., Alzheimer’s, dementia, epilepsy, and Parkinson’s) through SARs and bioinformatics data analysis is now seen as an essential tool towards their treatment [68]. However, despite experimental advances and the resulting greater and more accurate data provided, computational modeling approaches, such as SAR, also encounter challenges when studying the CNS, including:

i. A single activity resulting in/from multiple receptor signals [38].
ii. Multiple mechanisms (e.g., reuptake inhibition and release) of drug-transporter complexes [69].
iii. Unexpected SARs of some phenethylamines (entactogens) [9].
iv. Legal restrictions.
v. Diversity of reported experimental in vitro data.
vi. High stereoisomer-dependent activity [70,71,72].
vii. Lack of experimental X-ray crystal structures [42].

Structure-activity relationship (SAR) methods have become a legitimate and valuable part of toxicology since the mid-1970s [73,74] and an extremely important part in the twenty-first century, as Cramer et al. published the first modern approach (intended primarily to prioritize structures for experimental evaluation) [75]. These methods are various forms of mathematical or statistical models that seek to predict the adverse biological effects of chemicals based on their structure. The prediction may be either of a qualitative (carcinogen/noncarcinogen) or quantitative nature, with the second group usually being denoted as Quantitative Structure-Activity Relationship (QSAR) models [76]. QSAR modeling is a well-established computational methodology for chemical data analysis. QSAR models are developed by establishing empirical, linear, or non-linear relationships between values of chemical descriptors computed from molecular structure and experimentally measured properties or bioactivities of those molecules, followed by applying these models to design novel compounds with desired properties [54,77]. QSAR studies attempt to explain ‘why certain structural features influence the actions of a given series of agents. Once SAR studies have been conducted, a QSAR study can be performed using correlational analysis to identify if action within a series of agents might be related to one or more specific physicochemical properties of the altered substituent. Measures include, but are not limited to, electronic character, spectroscopic data, steric size, overall or specific shape, and lipophilicity [78]. SAR computational studies related to the CNS have been performed and appeared in the literature for quite some time [29,30,73]. Unfortunately, however, modern QSAR studies, in comparison, are still in their infancy regarding CNS-related studies, as illustrated by the comparatively lower publication-related numbers as shown in Figure 3 [74]. Increased use of modern QSAR methods, with their more advanced features, for CNS studies could help address and/or overcome many of the challenges listed above and beyond.

In 1983, Gupta et al. [79] published arguably one of the first reviews of QSAR studies of hallucinogens. More specifically, they combined small-scale database results to provide a comprehensive perspective on the explanation of hallucinogenic activity. They concluded that for phenalkylamines, their electronic and hydrophobic properties are the most critical molecular properties for psychedelic activity. They also discussed some inconsistencies in some of the reported data, such as the fact that some of the compounds in the dataset had hallucinogenic activity while others did not. This may be due to the fact that there is potentially more than one target for hallucinogenic activity, and structurally/conformationally different molecules might bind different targets for different reasons (e.g., one may be driven by electronic properties and the other by hydrophobic). As a result, it was further suggested that in such cases, the tautomers should be considered very carefully. However, as noted, one of the challenges faced by the datasets available at that time was their relatively small size. Hallucinogenic activity is a result of multiple drug-target (receptor and transporter) interactions, and if the response (experimental data) is an adverse outcome rather than a single biochemical key event, QSAR may fail, especially if the dataset is relatively small [80]. In classical QSAR, only one predicted activity is modeled at a time. In contrast, in drug development, multiple activities, both on- and off-target, are needed for prioritizing compounds. The set of techniques for prioritizing compounds based on more than one predicted activity simultaneously is called multi-parameter optimization or multi-task modeling. In general, this objective can be achieved by an ensemble of single-task models or by a single model that can predict more than one activity simultaneously using non-neural or neural net-based techniques, including deep learning, which has become popular in recent years [77,81]. Instead of hallucinogenic activity, each essential biological step should be considered a response to being able to create a consistent model. It is noted that a newer approach that has been used successfully is three-dimensional quantitative structure-activity relationships (3D-QSAR). Recently, Ma et al. [82] developed and used advanced predictive 3D-QSAR models to design and modify compounds that target two specific receptors in the brain: the dopamine D3 receptor (D3R) and the serotonin receptor (5-HT1AR). Their dataset consisted of 39 compounds, and the models they created showed excellent accuracy in predicting the activity of these compounds on the receptors. The external predictability of the models was found to be high, indicating their ability to accurately predict the activity of new compounds. For the D3R models, the predictability coefficient (r2 pred) was 0.811, while for the 5-HT1AR models, it was 0.869. The statistical robustness of the models was also evaluated, and they demonstrated good performance. The coefficients of determination (r2) for the D3R models were 0.967, indicating a strong correlation, and for the 5-HT1AR models, it was even higher at 0.995. The models also showed reasonable values for the cross-validated squared correlation coefficient (q2), which assesses the reliability of the predictions. For the D3R models, q2 was 0.635, and for the 5-HT1AR models, it was 0.514. Overall, these results indicate that the developed 3D-QSAR models are robust and accurate tools for designing and modifying compounds that target these specific receptors, providing valuable insights for drug discovery and development.

The complexity of a ligand or drug interacting with multiple targets can, however, also be examined and useful insights gained. For example, amphetamine and its derivatives exhibit various pharmacological activities, including psychostimulant, hallucinogenic, entactogenic, anorectic, or antidepressant. Furthermore, the mechanisms of action underlying these effects are usually related to the ability of different amphetamines to interact with a diverse variety of MATs or receptors. However, many of these same compounds are also potent and selective MAO inhibitors. MAO is the main catabolic enzyme for biogenic monoamines (NE: norepinephrine; DA: dopamine; 5-HT: 5-hydroxytryptamine; β-phenethylamine, tyramine, and benzylamine). MAO exists in two isoforms (isozymes), MAO-A and MAO-B; both are outer mitochondrial membrane-bound flavoproteins [83]. Consequently, to date, a significant portion of QSAR studies conducted in the CNS field have in fact focused on MAO inhibition.

One of the early studies in this field was conducted in 2002 by Vallejos et al. [84], where they investigated the interaction between phenylisopropylamine compounds and the enzyme monoamine oxidase A (MAO-A). Specifically, they focused on evaluating the effect of charge-transfer interactions by calculating the highest occupied molecular orbital (HOMO) energies and charges on the aromatic rings of two sets of MAO-A inhibitors. One set contained compounds with 4-amino substituents.

To assess the relationship between molecular properties and inhibitory activity, multiple-linear regression analysis was performed using experimental inhibition values (IC50) and the calculated descriptors for a dataset consisting of 33 compounds from the two sets, as well as separately for each set. The findings of Vallejos et al. suggested that compounds with electron-rich aromatic rings and higher HOMO energy levels exhibited increased potency in inhibiting MAO-A within their dataset. This observation indicates that charge-transfer interactions and HOMO energy levels play significant roles in determining the inhibitory activity of these compounds.

Furthermore, the relationship between HOMO energy and MAO-A inhibition may also have relevance in understanding the compounds’ hallucinogenic activity. Although Vallejos et al.’s study did not elaborate on this aspect, it is worth noting that HOMO energy can influence the interaction of molecules with their target receptors in the brain, potentially affecting their hallucinogenic effects. Exploring the correlation between HOMO energy and hallucinogenic activity could provide valuable insights into designing novel compounds with desired psychedelic properties.

More recently, Reyes-Prada et al. [83] conducted a comprehensive review of MAO inhibition by amphetamines and its potential impact on pharmacology. In their article, they discussed both experimental and computational findings, including docking studies. The focus was on understanding how the structural properties of amphetamines influence their ability to inhibit MAO. Among their conclusions, Reyes-Prada et al. suggested that S-enantiomers are more effective in inhibiting MAO. However, their review did not provide explicit indications of any interconnection between HOMO energy and MAO inhibition.

These studies collectively emphasize the ongoing efforts to investigate the intricate relationship between molecular properties, such as charge-transfer interactions, HOMO energy levels, and the inhibitory activity of compounds targeting MAO-A. The research contributes to our understanding of the underlying chemistry behind drug-receptor interactions, providing insights that can aid in the design of novel compounds with improved inhibitory activity and potential therapeutic applications.

Chirality as it pertains to the properties and roles of ligands and therapeutic drugs has attracted considerable attention, especially in neurochemistry [85]. For instance, different enantiomers have been shown to exhibit significantly different activities toward biological targets, including those involved in the CNS [86]. Amphetamine MDMA (3,4-methylenedioxymethamphetamine) is one such species, with R- and S-MDMA being reported to exhibit different activities towards CNS targets [72,86,87,88]. For example, Fresqui et al. [89] have used QSAR to investigate the influence of *R*- and *S*-enantiomers (stereoisomers) of a range of amphetamines on MAO. More specifically, they generated two QSAR models, one for each set of stereoisomers, to understand the parameters best able to model stereoselective activity. As a result, they obtained a multivariate QSAR model for a set of 34 amphetamine derivatives with *R*- and *S*-enantiomers. They concluded in part that within the active site, the ligands interact with the enzyme via π–π stacking interaction with Tyr407 and an inclined face-to-face interaction with Tyr444. In addition, however, unlike the *S*-configurations, the *R*-enantiomers also preferred to interact via aromatic hydrogen-hydrogen interactions with Tyr197.

**Figure 7:**
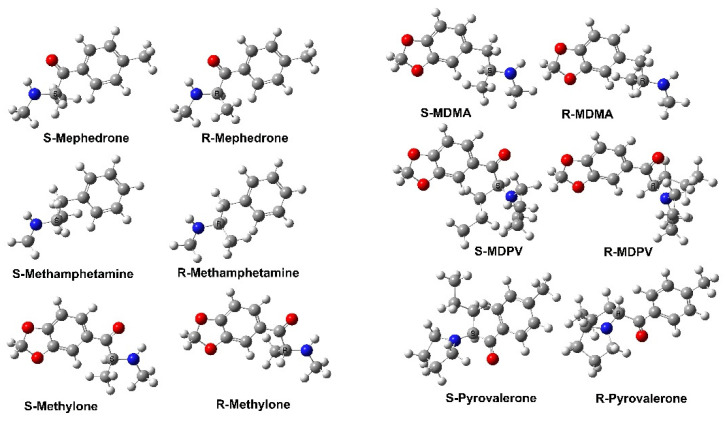
The molecular structures, showing their stereoisomers, of several important cathinones and amphetamine analogs (R-MDMA docked within the central binding site of SERT) are provided in Figure S1.

Designer drugs are synthetic compounds developed to provide rewarding effects like illicit drugs of abuse while circumventing existing legislative classification and penalties [90]. Synthetic cathinones (Figure 7) are oxidized keto-analogs of amphetamines and are one of the most prevalent classes of such drugs. Indeed, after amphetamines, they are the second-largest group of designer drugs. Glennon and Dukat have published an extensive summary of the SAR of cathinones [74]. More specifically, they analyzed the SAR properties of 63 compounds in detail and concluded that the cathinones exhibit stereoselective activity like that of MDMA. For example, S (-)-cathinone was more potent than its R (+)-enantiomer in most cases. In addition, some agents also displayed releasing action at NET and/or SERT. Subsequently, MDPV (Figure 7) was identified as a drug of abuse that acted primarily as a reuptake inhibitor at DAT. While most SAR studies on synthetic cathinones have focused on DAT and SERT actions, this study further supports a role for NET. It is noted that several other studies have also been reported that have allowed for a general SAR to be formulated [74].

Presently, there is considerable experimental data available to researchers that will greatly increase in size and reliability with advancements in technology and techniques. For example, there are 97,622 Ki data for multiple CNS targets (e.g., receptors and transporters) available in one Psychoactive Drug Screening Program (PDSP) [91] database. This presents a tremendous opportunity for the use of computational approaches, either on their own or in combination with experiments, to have a significant impact on relevant CNS-related investigations.

In recent studies, the application of field-based 3D-QSAR modeling has emerged as a valuable approach for understanding biological activity and designing new molecules in the field of psychedelic chemistry. Catalani et al. [92] employed 3D QSAR models, specifically the 3D-field QSAR and RVM (relevance vector machine) models, to predict the biological activity of designer benzodiazepines (DBZDs) on the γ-aminobutyric acid A receptor (GABA-AR). The models exhibited excellent performance statistics, indicating their reliability in predicting the potency of DBZDs. Furthermore, the authors conducted scaffold hopping studies, revealing the potential for improving the biological activity of DBZDs by replacing the pendant phenyl moiety with a five-membered ring. This finding suggests the existence of an unexplored chemical space for DBZDs, highlighting the significance of computational techniques in expanding the design possibilities for novel psychedelic compounds [92]. Additionally, Floresta and Abbate [93] presented a comparative analysis between machine learning approaches and field-based 3D-QSAR models for predicting the affinity of substances acting on the serotonin 2A receptor (5-HT2AR). The resulting QSAR models demonstrated promising predictive and descriptive capabilities, enabling the investigation and identification of unclassified molecules. By combining the best-performing machine learning algorithm and the classical field 3D-QSAR model, a consensus model was developed, which facilitated the exploration of 5-HT2AR activity in a vast library of natural products and the classification of recently reported new psychoactive substances [93]. These studies collectively demonstrate the value of field-based 3D-QSAR modeling and its integration with machine learning techniques in elucidating the activity and design of psychedelic compounds, providing researchers with valuable tools for drug discovery and classification in the rapidly evolving field of psychedelics.

Further details on performing computer-aided drug design have been published in several recent reviews [94,95], while a text summary is also provided in the Supporting Information (Table S1).

### Possible Future Developments

Artificial intelligence (AI) has emerged as a powerful tool within the realm of computational modeling. In particular, AI techniques such as machine learning and deep learning have enabled researchers to extract valuable insights from complex and heterogeneous datasets, accelerating the discovery and development of novel psychedelic drugs. By leveraging its algorithms, researchers can analyze large-scale genomic data, in vivo and in vitro drug test results, and other diverse sources of information to uncover patterns, relationships, and predictive models [96,97,98,99]. Consequently, AI-driven computational approaches have the potential to streamline the design and optimization of new psychedelic compounds by predicting their properties, interactions, and potential therapeutic effects. Moreover, AI-driven informatics tools have facilitated integrating and interpreting disparate data sources, allowing for a more comprehensive understanding of mental health disorders and the development of targeted treatments. The utilization of AI in psychedelic chemistry showcases its transformative potential in advancing research in the Central Nervous System. It underscores its importance in shaping the future of drug discovery and development.

## 4. Conclusions

In this minireview, we discussed the application of in silico methods to gain molecular insights into critical aspects of the CNS. In particular, the focus was on using these methods to gain insight and a deeper understanding of ligand/drug interactions and their targets, with the goal of designing and developing more effective and specific therapeutics based on amphetamines and entactogens.

As mentioned earlier, most scientific literature in the field of CNS therapeutics has used in vitro experimental approaches to gain meaningful insights. Recent promising developments in hardware and software technologies have made it an achievable and fruitful goal to leverage complementary and disparate computational approaches and studies to provide deeper insights and/or predict chemical properties and kinetics. This brief overview uses examples from the literature to illustrate the usefulness of computational methods in silico for studying biomolecular systems and complexes and drug design and development. In particular, we show that the prospective use of docking can provide essential insights into ligand binding to active and allosteric sites. These can be enhanced by the complementary use of MD to refine docking positions further and obtain more reliable binding energies. As shown, multiscale computational studies using quantum mechanical (QM) chemical cluster models and/or QM/MM approaches can provide information at the electronic level. Thus, a more comprehensive understanding of key biomolecules (e.g., receptors) and/or their interactions with ligands (e.g., drugs) can be obtained. In addition, studies have shown that the increasing availability of accurate experimental and computational data is helping to improve the utility and predictive power of structure-activity relationship (SAR)-based informatics methods. Considerable databases can now be built to develop reliable QSAR and even 3D QSAR models.

Combining multi-scale (Figure 4) computational methods (Docking, MD, QM/MM, QSAR) with experimental data is of paramount importance in obtaining reliable molecular-level information about CNS and mental health problems. The intricate complexities of these systems necessitate a holistic approach that bridges the gap between theoretical models and real-world observations. By integrating computational simulations that span multiple lengths and time scales with experimental data, researchers can unravel the underlying mechanisms governing CNS function and the intricate etiology of mental health disorders. This synergistic approach not only enhances our understanding of these intricate systems but also enables the identification of novel therapeutic targets and the development of more effective treatments. Furthermore, the integration of multi-scale computational methods with experimental data facilitates the exploration of the intricate interplay between genetic, environmental, and physiological factors, ultimately paving the way for personalized medicine and improved patient outcomes in the realm of mental health.

Despite their increasing usage and potential, the application of in silico approaches for studying CNS-related bioactivity and chemistry also faces challenges, such as limited experimental data and/or required computational resources. However, the examples illustrate how the innovative application of in silico methods is an advancing area of impact and opportunity in the neuropharmaceutical and biotechnology industries. In addition, they will be needed to address the current and future challenges facing the related research fields and, notably, our global society. It is our belief that the present mini-review should help computational and experimental researchers interested in or engaged in this challenging but enriching field enhance their studies.

## Data Availability

Not applicable.

## Supplementary Materials

The following supporting information can be downloaded at: https://www.mdpi.com/article/10.3390/molecules28165966/s1, Figure S1: R-MDMA docked in the central binding site of Serotonin (SERT). Structure of SERT taken from the protein database (PDB ID: 7LIA); Table S1: An overview, step-by-step guide, to performing computer-assisted drug discovery (CADD), particularly in psychedelic chemistry.
