# Supplementary material for: Applications and Potential of In Silico Approaches for Psychedelic Chemistry
Source: Molecules. 2023 Aug 9;28(16):5966. doi: 10.3390/molecules28165966 (PMC10459288; doi:10.3390/molecules28165966)
Supplement: Supplementary file 1 [file molecules-28-05966-s001.zip › molecules-2485272-supplementary.pdf]

Applications and Potential of In Silico Approaches for Psychedelic Chemistry

Supporting Information

(Total pages: 3; Figure S1 and Table S1)

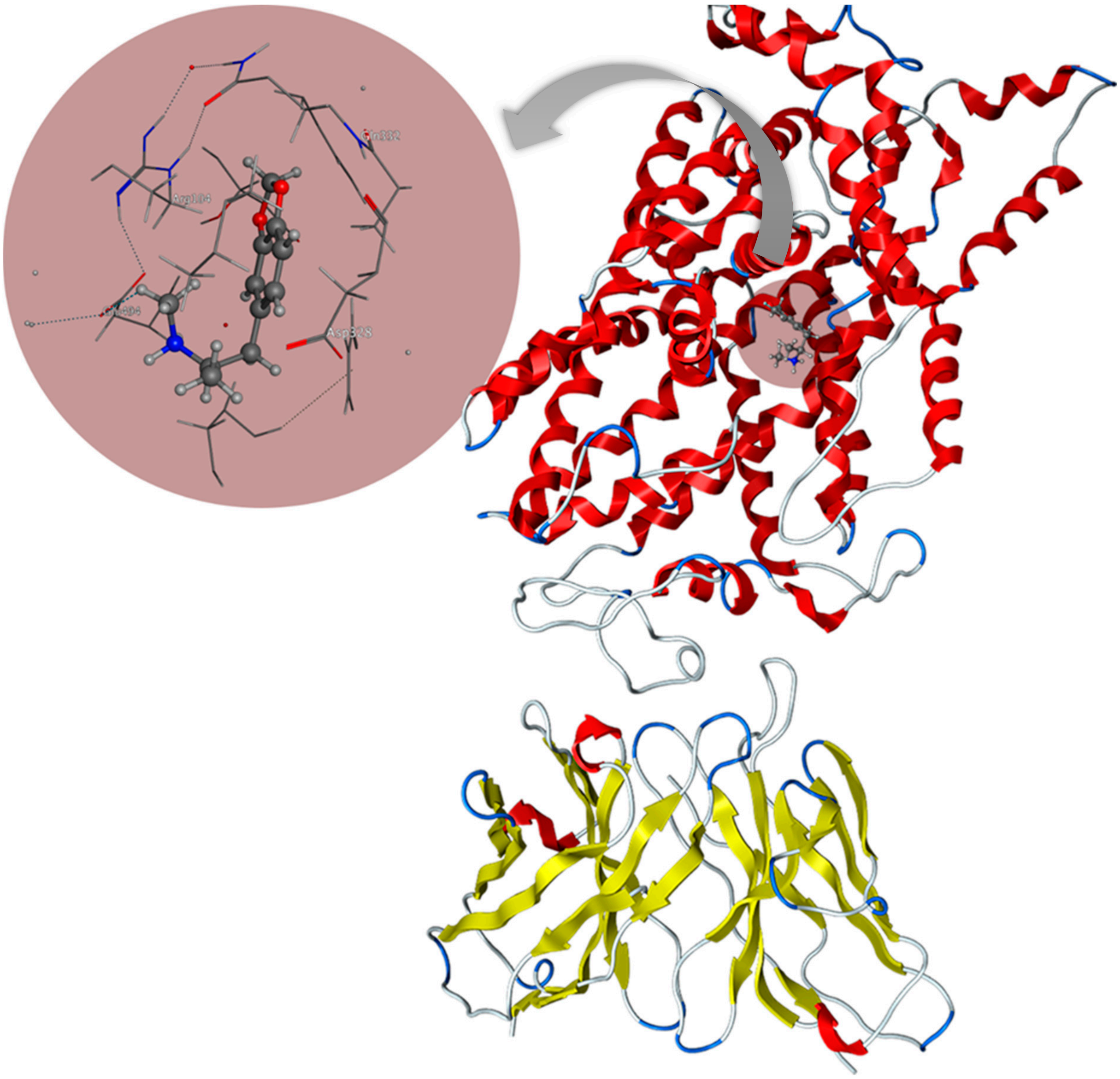

Figure S1: R-MDMA docked in the central binding site of Serotonin (SERT). Structure of SERT taken from the protein database (PDB ID: 7LIA).

Table S1. An overview, step-by-step guide, to performing computer-assisted drug discovery (CADD), particularly in psychedelic chemistry.

(CADD is a process involving using computational methods and techniques to aid in the identification and design of potential drug candidates.)
**Defining the Objective**: Clearly state the objectives of your drug discovery project. Understand the target or disease you aim to treat and determine if there are any known psychedelic compounds that might be relevant.
**Data Collection and Curation**: Gather relevant data, including chemical databases, literature, and experimental results related to psychedelic compounds. Curate and validate the data to ensure its quality and relevance.
**Target Identification and Validation**: Identify the specific molecular target that you want to interact with using the psychedelic compound. This could be a receptor, enzyme, or other biological molecules associated with the desired therapeutic effect. Validate the target's role in the disease or condition of interest.
**Virtual Screening**: Use computational methods like molecular docking or molecular dynamics simulations to perform virtual screening of psychedelic compounds against the identified target. This step helps in predicting how well the compounds might interact with the target.
**Lead Compound Selection**: Based on the virtual screening results, select a set of lead compounds that show promising interactions with the target. These compounds will serve as the starting point for further optimization.
**(Quantitative) Structure-Activity Relationship (QSAR) Analysis**: Analyze the relationships between the chemical structures of the lead compounds and their biological activities. This information will guide the modification of the compounds to improve their potency and selectivity. If possible, a quantitative (cor)relation between activity and property would accelerate and automize the process.
**Computational Chemistry and Design**: Utilize quantum mechanics calculations, molecular modeling, and other computational techniques to optimize the lead compounds. This process involves tweaking the chemical structure of the compounds to enhance their binding affinity and pharmacokinetic properties.
**ADME/Tox Prediction**: Predict the Absorption, Distribution, Metabolism, Excretion (ADME), and toxicity properties of the optimized compounds using computational models. This step helps in identifying potential issues that could arise during the later stages of drug development.
**Synthetic Feasibility Analysis**: Assess the synthetic feasibility of the designed compounds. Consider factors like synthetic pathways, availability of starting materials, and overall cost-effectiveness.
**In Silico Pharmacology:** Predict the pharmacological profile of the optimized compounds, including their potential therapeutic effects and side effects, using computational models and databases.
**Hit-to-Lead Optimization**: Further refine the compounds based on ADME/Tox prediction, pharmacology, and synthetic feasibility analysis results. Iteratively optimize the chemical structures to achieve desired properties.
**Biological Assays and In Vitro Testing**: Synthesize the most promising compounds and test them in vitro against the target and disease models. Validate their biological activities and assess their potency and selectivity.
**Animal Studies (In Vivo Testing)**: If appropriate and ethical, perform animal studies to evaluate lead compounds' pharmacokinetic and pharmacodynamic properties in living organisms.
**Hit Validation and Candidate Selection**: Analyze the results from in vitro and in vivo testing to validate the hits and select the most promising candidate(s) for further development.
**Iterative Optimization and Preclinical Studies**: Continuously optimize the lead compound(s) based on the results of preclinical studies, including safety and efficacy evaluations.
Remember that drug discovery is a complex and iterative process. Computational methods play a crucial role in identifying potential drug candidates, but experimental validation is essential before advancing candidates to clinical trials and eventual market approval.
